# Synthesis and Property Analysis of a High-Temperature-Resistant Polymeric Surfactant and Its Promoting Effect on Kerogen Pyrolysis Evaluated via Molecular Dynamics Simulation

**DOI:** 10.3390/polym17152005

**Published:** 2025-07-22

**Authors:** Jie Zhang, Zhen Zhao, Jinsheng Sun, Shengwei Dong, Dongyang Li, Yuanzhi Qu, Zhiliang Zhao, Tianxiang Zhang

**Affiliations:** 1 CNPC Engineering Technology R&D Co., Ltd., Beijing 102206, China; 2 CNPC Greatwell Drilling Co., Ltd., Beijing 124011, China

**Keywords:** polymeric surfactant, shale gas, kerogen, molecular dynamics

## Abstract

Surfactants can be utilized to improve oil recovery by changing the performance of reservoirs in rock pores. Kerogen is the primary organic matter in shale; however, high temperatures will affect the overall performance of this surfactant, resulting in a decrease in its activity or even failure. The effect of surfactants on kerogen pyrolysis has rarely been researched. Therefore, this study synthesized a polymeric surfactant (PS) with high temperature resistance and investigated its effect on kerogen pyrolysis under the friction of drill bits or pipes via molecular dynamics. The infrared spectra and thermogravimetric and molecular weight curves of the PS were researched, along with its surface tension, contact angle, and oil saturation measurements. The results showed that PS had a low molecular weight, with an MW value of 124,634, and good thermal stability, with a main degradation temperature of more than 300 °C. It could drop the surface tension of water to less than 25 mN·m^−1^ at 25–150 °C, and the use of slats enhanced its surface activity. The PS also changed the contact angles from 127.96° to 57.59° on the surface of shale cores and reversed to a water-wet state. Additionally, PS reduced the saturated oil content of the shale core by half and promoted oil desorption, indicating a good cleaning effect on the shale oil reservoir. The kerogen molecules gradually broke down into smaller molecules and produced the final products, including methane and shale oil. The main reaction area in the system was the interface between kerogen and the surfactant, and the small molecules produced on the interface diffused to both ends. The kinetics of the reaction were controlled by two processes, namely, the step-by-step cleavage process of macromolecules and the side chain cleavage to produce smaller molecules in advance. PS could not only desorb oil in the core but also promote the pyrolysis of kerogen, suggesting that it has good potential for application in shale oil exploration and development.

## 1. Introduction

China is rich in continental lacustrine shale oil resources, producing 340 × 10^4^ t in 2022 [1]. According to an estimation by the Ministry of Natural Resources, the potential recoverable resources in China are 34.98 × 10^8^ t [2]. Shale oil reservoirs are mainly composed of shale and oil shale. A small amount of shale oil exists in a free state in porous media, while the vast majority is adsorbed in organic polymers such as kerogen. Oil shale is a sedimentary rock in which organic matter is embedded, known as kerogen [3]. Kerogen is the source of hydrocarbon components in oil shale reservoirs and has complex chemistry [4]. Pyrolysis is the most effective way to convert organic kerogen in oil shale into valuable oil and natural gas. Research showed that the effect of pyrolysis on kerogen increased with temperature, while it was inhibited by excessively high temperatures. The pyrolysis temperature range of kerogen was reported to be higher than 2000 K [5,6]. Therefore, researchers have developed catalysts to reduce the pyrolysis temperature of kerogen, such as inorganic salt [7], transition metal salt [8], and montmorillonite [6]. In addition, the research on shale oil’s adsorption, desorption, and diffusion motion is still in its infancy due to the low permeability of tight shale. The behavior of kerogen under pyrolysis during the heating process is usually studied through molecular simulation.

Furthermore, the low permeability and porosity of shale formations increase the difficulty of exploration and development [9]. Surfactants are a common agent for the exploration and development of shale oil. For example, the surfactants in drilling fluids include cationic and nonionic surfactants and their hydrophilic end can adsorb onto clay through electrostatic forces, hydrogen bonds, and other interactions. In contrast, their hydrophobic end forms a hydrophobic layer on the surface of clay, inhibiting its hydration expansion [10,11]. Surfactants with a strong foaming ability are used less frequently due to their negative effects on the rheology of drilling fluids during drilling. In addition, surfactants, such as nonionic cationic, anionic, and amphoteric, have been shown to reduce the surface tension and change the wettability of formation surfaces to enhance the recovery rate of shale oil and gas [12,13,14]. Their performance was affected by the formation temperature, salinity, pH value, and other factors. For example, high temperatures will affect the overall performance of the surfactant, resulting in a decrease in its activity or even failure. However, the effect of such surfactants on the pyrolysis behavior of kerogen has rarely been studied.

In this study, a high-temperature-resistant polymeric surfactant (PS) was synthesized through emulsion polymerization and its structure, thermal stability, and relative molecular weight were observed. The surface tension of the PS was measured in various media and at different temperatures. Its effect on the wettability and oil saturation of the shale core was also evaluated. In addition, a three-dimensional molecular model of kerogen and PS was established from the microscopic atomic level through molecular simulation methods using Materials Studio software 2021. The ReaxFF reaction molecular dynamics simulation method was used to explore the cracking mechanism of kerogen molecules after the addition of PS, providing theoretical guidance for developing new enhanced oil recovery agents.

## 2. Experimental Sections

### 2.1. Materials

Perfluorohexyl ethyl alcohol, pentadecyl acrylate, azobisisobutyronitrile, and perfluorooctylethyl acrylate were obtained from Sinopharm Chemical Reagent Co., Ltd., Shanghai, China. Sodium dodecyl sulfate, octadecyl methacrylate, and dodecyl mercaptan were purchased from Shanghai Macklin Biochemical Co., Ltd., Shanghai, China.

### 2.2. Preparation of PS

Perfluorohexyl ethyl alcohol, sodium dodecyl sulfate and dodecyl mercaptan were dissolved in deionized water and stirred for 15 min. Pentadecyl acrylate, octadecyl methacrylate, and perfluorooctylethyl acrylate (with relative contents of 41%, 8% and 32% of each monomer) were added to the solution and stirred evenly for 15 min under nitrogen conditions. The temperature of the mixture was raised to 60–70 °C. The azobisisobutyronitrile was used to initiate the system′s polymerization and the reaction time was 3 h. Finally, the polymeric surfactant (PS) was obtained.

### 2.3. Experimental Methods

The PS powder was obtained by washing the emulsion with a large amount of ethanol, centrifuging and then drying at 60 °C for 50 h. The infrared spectrum of PS was measured using a Nicoler IS10 Fourier Transform Infrared (FTIR) spectrometer (Thermo Nicolet Corporation, Waltham, MA USA).

A synchronous thermal analysis apparatus (STA449 F5 Jupiter, Netzsch, Waldkraiburg, Germany) was used to evaluate the thermal ability of the PS under a nitrogen flow at 50–700 °C, with a rate of 10 °C/min.

The relative molecular weight of the PS was determined via gel permeation chromatography (Malvern Viscotek 3580, Malvern, UK). The solvent and mobile phase were 1,2,4-trichlorobenzene (containing 0.025% antioxidant 2,6-dibutyl-p-cresol), with a column temperature of 150 °C and a 1.0 mL/min flow rate. The polystyrene standard sample was used for universal calibration.

The surface tension of the PS was evaluated in various media at room temperature after aging using a Sigma701 surface tension meter (Bai Olin, Stockholm, Sweden). The experiment utilized the ring method for surface tension measurement, with the following standardized procedure: First, the sample chamber was filled with deionized water and securely mounted on the instrument base. The base was elevated to fully immerse the platinum ring into the liquid. The base was lowered at a constant rate upon initiating the test protocol. Calibration was completed by measuring the surface tension of the water when the platinum ring detached from the liquid surface. PS solutions were prepared at concentrations spanning 0.1–0.5 wt% and were aged for 16 h at 25–150 °C using a roller furnace (Hengtaida, Qingdao, China). The sample was poured into the sample chamber, and the base was raised to submerge the platinum ring. The measurement cycle was activated, and the base was steadily lowered to quantify the interfacial tension at the oil-water boundary. Replicated measurements (3–5 times) were conducted under identical experimental conditions. Data reliability was ensured by calculating the arithmetic mean of repeated determinations.

The rock cores were removed from the shale oil well in the Permian Lucaogou Formation block of Jimsar Sag. The block was composed of rocks deposited by lacustrine factors [15]. Their whole rock mineral and clay content was measured using a D8 Advance X-ray diffractometer (Bruker, Bremen, Germany).

The contact angles of the rock cores were measured using an OCA50 contact angle meter (DataPhysics, Filderstadt, Germany). The sample was immersed in oil for 7 d. The contact angle of the dried core was measured and then immersed in a PS solution for 2 d. Finally, its contact angle was re-evaluated.

The rock core was first placed into a MicroMR12-040V nuclear magnetic resonance instrument (Newmai, Suzhou, China) to measure the energy spectrum. Then, crude oil was injected into the core under a high-pressure environment for 3 d, and then the energy spectrum was calculated for the second time. After the measurement, it was placed in a 0.5 wt% PS solution at room temperature for 7 d. Finally, its third energy spectrum was evaluated.

### 2.4. Molecular Simulation

According to the genetic types of sediments, the I-type lacustrine sedimentary origin kerogen structure model was used as the basis. The three-dimensional kerogen substructure model was optimized using the molecular dynamics (MD) method, and the simulation calculation process was based on maintaining its structural stability and minimum energy. Next, its structure was optimized, and the bond level distribution, bond length distribution, and electrostatic potential energy level of various chemical bonds in its molecular structure were calculated based on the optimization results, with a focus on analyzing the pyrolysis reaction of kerogen molecules [16].

## 3. Results and Discussion

### 3.1. Characterization

**Figure 1:**
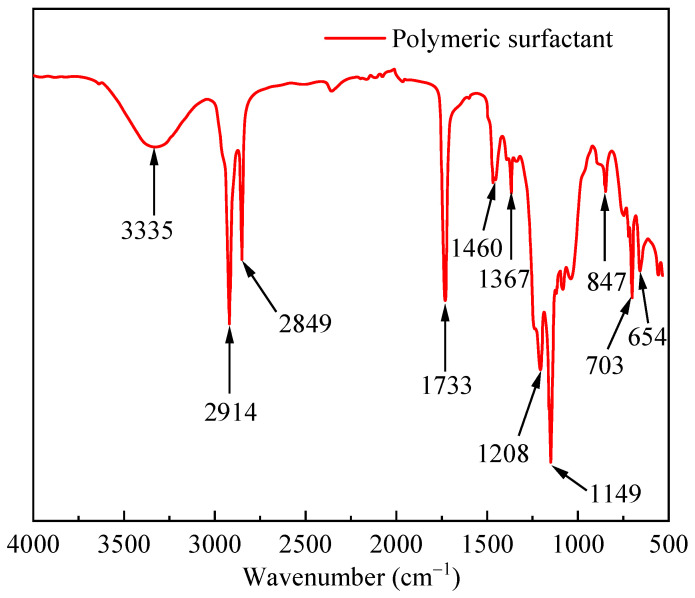
Infrared spectrum curve of PS.

Figure 1 shows the infrared spectrum of the PS. The stretching vibration peak at 3335 cm^−1^ was a hydrogen bond binding the hydroxyl group of the acrylic ester side chain with water. The peaks at 2914 cm^−1^ and 2849 cm^−1^ were attributed to methyl symmetric stretching vibrations. The characteristic absorption peak of the carbon–oxygen double bond was at 1733 cm^−1^. The deformation vibration peak of the methylene group was at 1460 cm^−1^. The peak at 1367 cm^−1^ was attributed to the symmetric bending vibration of the methylene group. The peaks at 1208 and 1149 cm^−1^ were attributed to the stretching vibration of the carbon-oxygen bond. The stretching vibration peak of the carbon–fluorine bond was at 847 cm^−1^. The stretching vibration peaks of the carbon–sulfur bond in the molecule were at 703 cm^−1^. The peak at 654 cm^−1^ was the out-of-plane bending of oxygen–hydrogen bonds.

**Figure 2:**
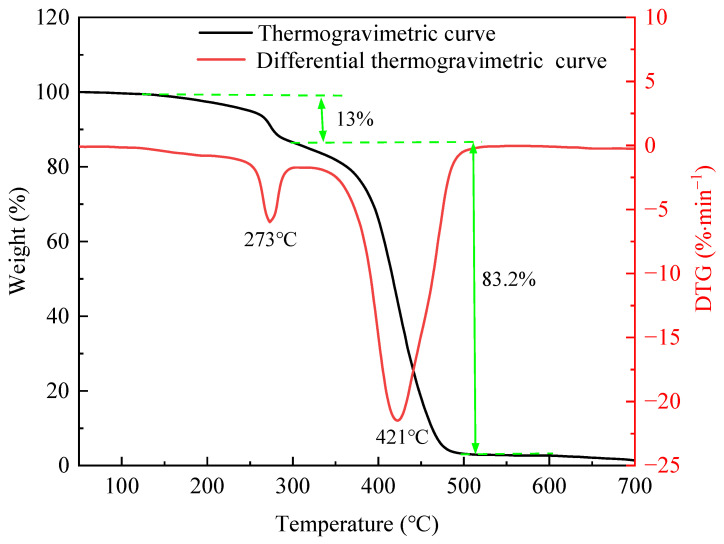
Thermogravimetric and differential thermogravimetric curves of PS.

Figure 2 shows the thermogravimetric and differential thermogravimetric curves of the PS. Its decomposition process included 50–125, 125–296, 296–498, and 498–700 °C. The mass of the PS decreased slightly in the first stage due to the volatilization of bound water. Its mass declined by 13% and the decomposed fastest at 273 °C in the second stage, owing to the breaking of side chains [17]. In addition, it dropped significantly by 83.2% in the third stage due to the breaking of the leading chains (C-C). Its fastest degradation temperature was 421 °C, indicating good thermal stability.

**Figure 3:**
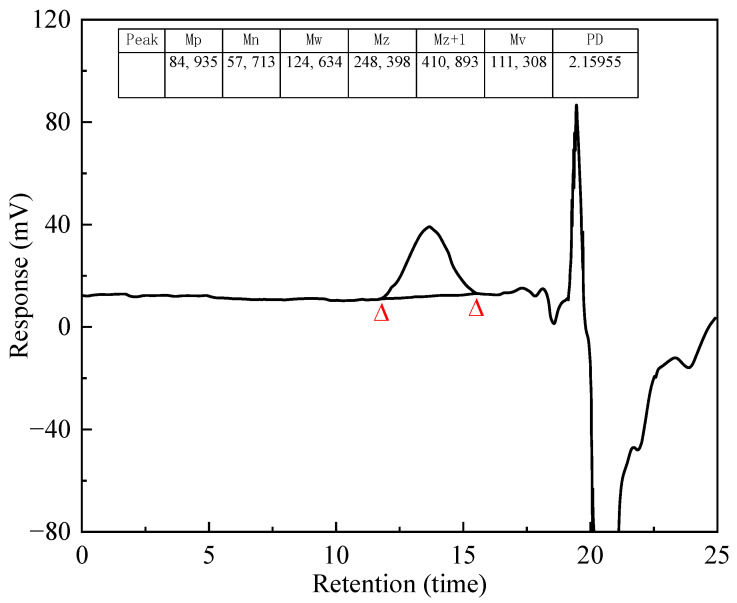
Molecular weight distribution of PS.

Figure 3 shows the relative molecular weight and distribution of the PS. The MW value was 124,634 and the PD value was 2.16. The synthesized PS had a high molecular weight and a narrow molecular weight distribution.

### 3.2. Surface Tension and Contact Angle Tests

**Figure 4:**
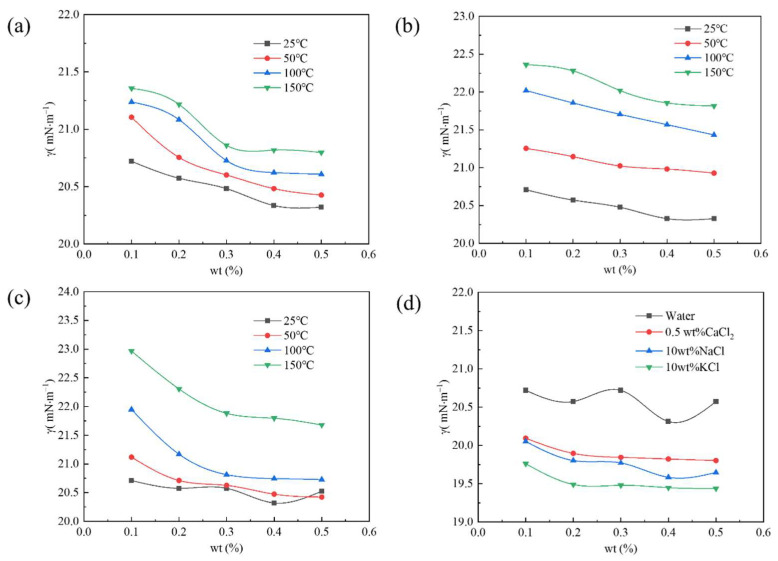
Change in surface tension with temperature of different concentrations of the PS in (**a**) water, (**b**) acid, (**c**) alkali, and (**d**) salt solutions.

Figure 4 shows the surface tension of the PS with different concentrations in various media changing with temperature. The PS with a mass fraction of 0.10–0.50 wt% could decrease the aqueous phase’s surface tension to less than 25 mN·m^−1^ after aging at 25–150 °C, as shown in Figure 4a. In addition, when its concentration was constant, its surface tension increased with temperature, and its increasing extent decreased with increase in concentration. At the same temperature, the surface tension of the PS gradually decreased and tended to become stable with an increase in concentration. At a specific concentration, the changing trend of the surface tension of the PS solution with a pH of 5 was the same as that under neutral conditions, but the increasing extent of surface tension was higher. The changing trend of the surface tension of the PS solution with a pH of 10 was similar to that under neutral conditions, as shown in Figure 4c. Although its surface tension increased significantly after aging at 150 °C, its surface tension values were still lower than 25 mN·m^−1^, indicating that PS in alkaline solution had the poorest temperature resistance. The addition of NaCl, KCl, or CaCl_2_ could enhance the surface activity of the PS and decrease the surface tension of the aqueous phase, as shown in Figure 4d. In addition, the surface tension of the PS in 10 wt% KCl was the lowest. These results could be attributed to the fluorine group, hydroxyl group, and silicon–oxygen chain of the PS forming a hydration layer in the aqueous phase and absorbing metal ions around the hydration layer through electrostatic interaction, resulting in a weakening of the electrostatic repulsion of the PS, enabling the PS molecules to become more closely arranged on the surface of the solution, and finally decreasing the surface tension [18,19,20].

**Figure 5:**
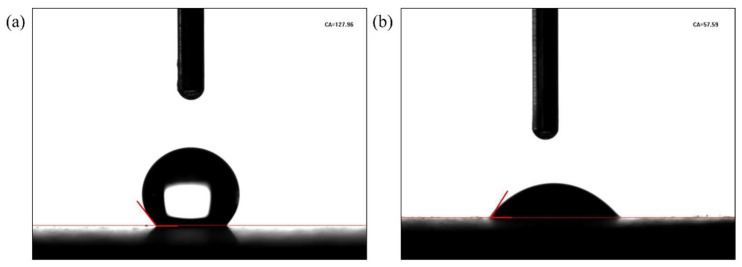
Contact angle of cores (**a**) before and (**b**) after being soaked in the PS solution.

The contact angles on the surface of shale cores before and after being soaked in the PS solution are shown in Figure 5. The contact angle of the core immersed in oil was 127.96°, suggesting that the core had reached an oil-wet state [21]. After immersing the core in 0.5 wt% PS solutions for 2 d, its contact angle was 57.59°, indicating that the core had reversed to a water-wet state. This was attributed to the ability of the PS to be absorbed in the core’s surface and change the rocks’ wettability.

### 3.3. Oil Saturation Measurement

The whole rock mineral and clay contents of the shale core are given in Table 1. Quartz, amorphous, and clay were the dominant internal mineral components, while a small amount of trace components such as dolomite, calcite, and pyrite were distributed in high-density minerals. The clay minerals were predominantly represented by illite.

**Figure 6:**
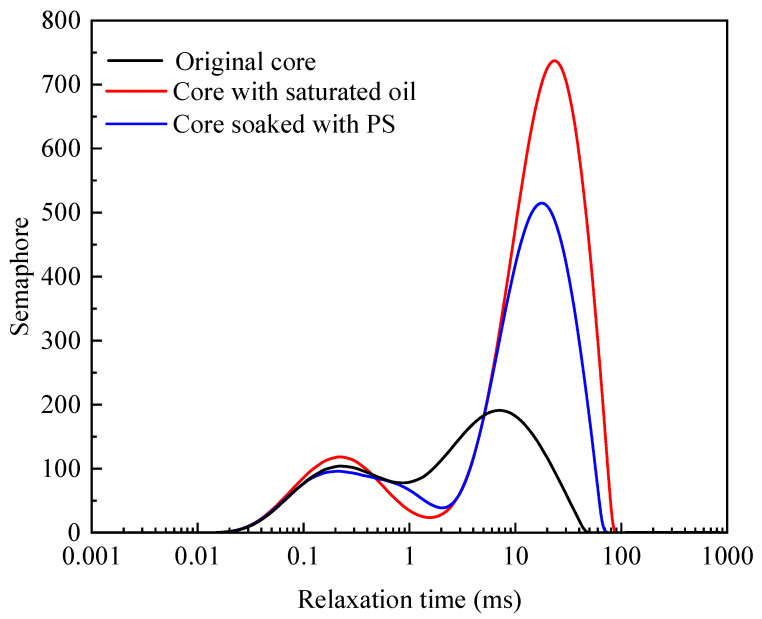
T2 mapping changes before and after soaking in the PS solution.

The shale core′s oil content in the saturated condition and the oil removal amount after soaking in the PS solution were measured using T2 mapping imaging technology. Figure 6 shows the semaphore change in shale cores before and after being soaked in the PS solution. In the T2 mapping, the chemical environment of hydrogen protons in the rock sample could be reflected by the relaxation time. The binding force and degree of freedom of hydrogen protons could be reflected by the changes in the spectrum, also considering the degree of hydrogen proton binding and the influence of the internal structure of the core column. After being soaked in the PS solution, the semaphore peak of the core, ranging from 3 to 90 ms, dropped significantly, indicating that the PS had good oil removal performance. The distribution of T2 mapping is directly related to the size of pores, while the effect of relaxation on proton diffusion is negligible.

The oil saturation of the shale core before and after soaking in the PS solution was calculated according to T2 mapping, and the results are given in Table 2. The oil saturation of the original core remained at 56.68%, and its saturated oil content accounted for 1.95% of its original weight. Its oil saturation was reduced to 25.63% after being soaked in the PS solution, indicating that the PS could promote oil desorption and have a good cleaning effect on the shale oil reservoir.

### 3.4. Simulation Results

**Figure 7:**
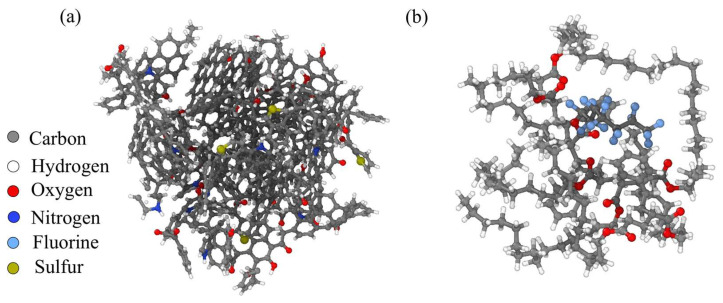
Molecular structure of (**a**) kerogen and (**b**) polymer surfactants. Colors correspond to the following atoms: C—gray; H—white; O—red; N—deep blue; F—light blue; S—earthy yellow.

Based on the structure model of lake-derived kerogen, a three-dimensional molecular structure model of kerogen was optimized using the molecular dynamics method. The three-dimensional structural characteristics of kerogen were further studied using Materials Studio software. Molecular dynamics calculations were performed using the Forcite module in Materials Studio software, and the hydrogen bonding energy of kerogen molecules was simulated using the Dreiding force field. The molecular dynamics method used classical Newtonian mechanics principles to explore the lowest energy configuration of a molecule by continuously changing the spatial geometric positions of atoms in the molecule. Using the widely used molecular model of kerogen and combining it with the constructed molecular fragments of kerogen, continuous optimization testing was carried out to obtain the molecular structure of kerogen, as shown in Figure 7a [22]. The chemical formula of a single kerogen molecule is C_853_H_601_O_45_N_13_S_4_. The PS is composed of water-containing styrene acrylic lotion, in which the mass ratio of water is 80%, and the mass ratio of styrene acrylic polymer is 20%, with a total of 464 atoms. Its chemical formula is C_162_H_271_O_16_F_15_, as shown in Figure 7b.

**Figure 8:**
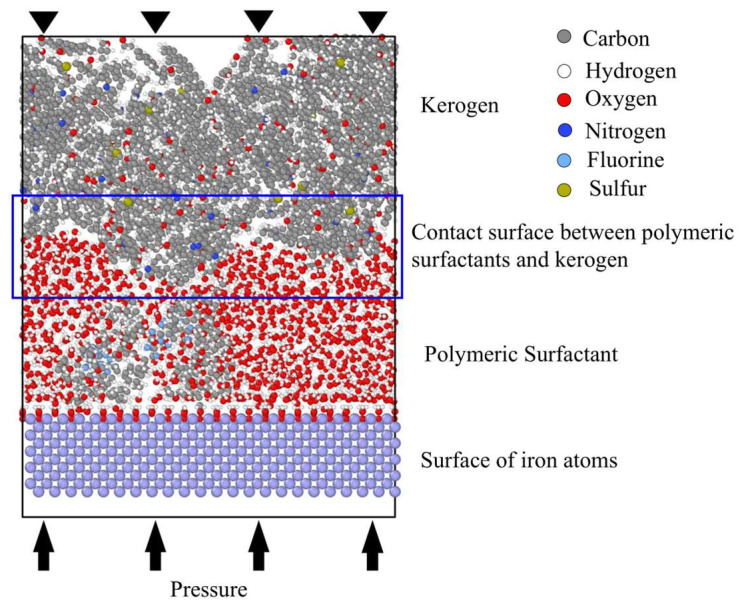
Initial architecture. Colors correspond to the following atoms: C—gray; H—white; O—red; N—deep blue; F—light blue; S—earthy yellow.

The initial structure of the simulation system is shown in Figure 8. The system was a cube with dimensions of 2.56 × 6.57 × 8.5 nm. It consisted of three main components: the molecular model of kerogen, the surfactants, and the metal surface of the drilling bit or string. The upper layer of the system contained four cheese root molecules filled with kerogen. The initial kerogen density was 1.0 g/mL, totaling 6064 atoms. Surfactants occupied the middle space of the system, between the kerogen molecules and the metal surface, containing 640 water molecules (1920 atoms) and 1 PS molecule (464 atoms). The metal surface in the lowest layer of the system was represented by iron crystals with a body-centered cubic structure, with a density of 7.9 g/mL. The model had periodic repeating features on the X and Y axes and remained intact on the Z axis (perpendicular to the metal surface direction).

The drilling fluid was between the drill bit (drill string) and the formation. Surfactants were added as part of the drilling fluid during drilling and production, attached to the surface of the bit; they played a role in balancing formation pressure, cooling, and lubricating the bit and string. The high-speed movement of the bit led to the movement of materials between the drilling fluid and the formation surface, causing formation fragmentation and cleavage of organic matter such as kerogen. Therefore, during simulation, the internal structure of the lowest metal layer was fixed in the model, and a uniform external force and constant linear velocity were applied to drive the model to operate. The surface agents and kerogen molecules were forced to move and evolve.

**Figure 9:**
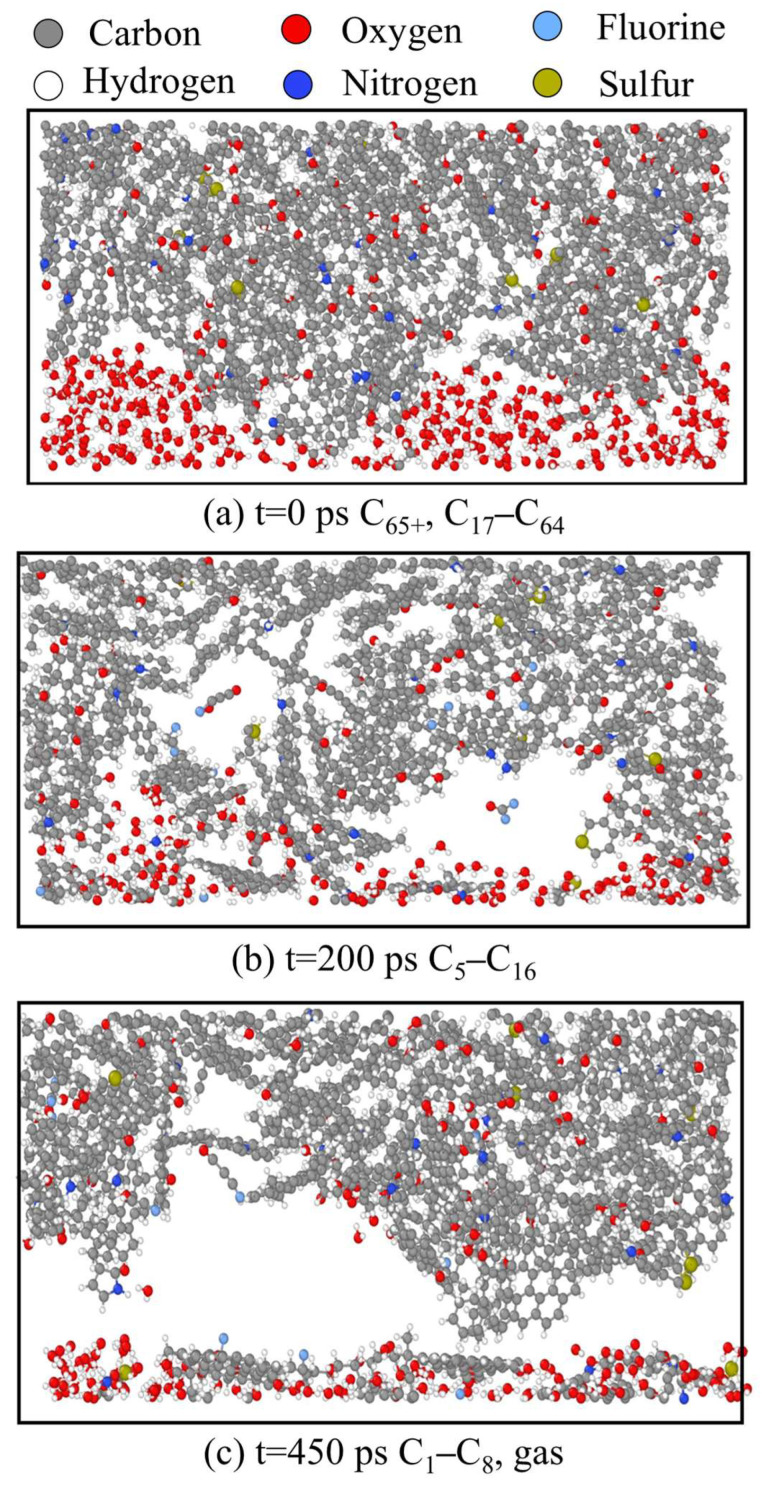
System structure at different time points on the interface between kerogen molecules and surfactants. Colors correspond to the following atoms: C—gray; H—white; O—red; N—deep blue; F—light blue; S—earthy yellow.

The motion trajectory and changes in the simulation system are shown in Figure 9. During the simulation process, the metal surface was in the bottom layer, which underwent shear motion at a constant velocity. The PS in the middle layer was indirectly driven as a working fluid. The topmost layer consisted of kerogen molecules that were relatively more difficult to drive. Shear and friction were formed between the PS and the kerogen, causing the kerogen molecules to gradually crack and produce light molecular fragments. The central reaction zone of the system was the interface between kerogen and the PS, where small molecules generated at the interface diffused towards both ends.

In the early stage of the simulation (0 ps), the system exhibited a tightly packed state with a straightforward interface between solid and liquid substances. At this stage, the kerogen molecules were mainly those with more than 65 carbon atoms, and some molecules between 17 carbon atoms and 64 carbon atoms (C_65+_ and C_17_–C_64_). In the middle stage of the simulation (200 ps), small and medium-sized molecules (C_5_–C_16_) were produced, and a small number of gas molecules were removed as the medium and large molecules were cracked, leaving a small number of voids. In the later stage of the simulation (450 ps), the kerogen molecules were broken down into small molecules (C_1_–C_8_) and released a large amount of gas that diffused towards both ends, leaving obvious voids.

In molecular dynamics simulations, a 500 ps equilibrium simulation was first performed to fully optimize the structures of various chemical substances and make the model more suitable for suit subsequent reaction simulations. The NPT ensemble was used in the equilibrium simulation, with a temperature of 298 K, a pressure of 1 bar, and an integration step size of 0.5 fs.

Under the NVT system, the integration step size was 0.25 fs, totaling 2,000,000 steps (500 ps). The external coupling temperature was 298 K and the coupling constant was 50 ps. The temperature of the simulated system varied freely due to friction and chemical reactions. During the simulation, the bottom metal surface moved horizontally at a uniform speed of 30 m/s.

The energy and force calculation was carried out using the ReaxFF reaction force field combined with the Fe/C/H/O/N/S/F force field parameter set to ensure accuracy. The dynamic charge balance algorithm (QEQ) was used to calculate the atomic charge.

**Figure 10:**
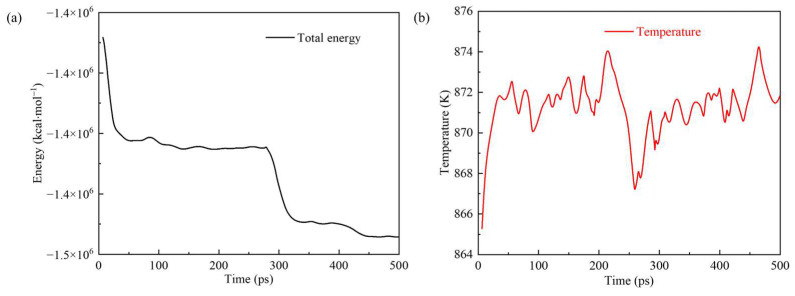
(**a**) Total energy and (**b**) temperature of the simulation system changing over time.

A simulated system’s thermodynamic properties can reflect the system’s total energy and other related properties. Figure 10 shows the total energy and temperature of the simulation system changing with time. The total energy continuously decreased step by step in the entire reaction process, indicating that the reaction was an exothermic process. The total energy decreased significantly within the time ranges of 0–50 ps and 250–300 ps due to the oxidation of the metal surface and the occurrence of violent reactions on the surface of kerogen molecules with molecular motion. During the two more extended periods of 50–250 ps and 300–500 ps, the total energy experienced a slow decline. In addition, the system’s temperature was always in the range of 870 ± 20 K.

**Figure 11:**
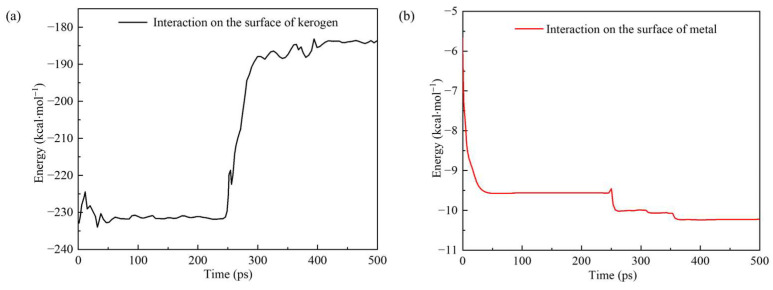
Variation in interaction energy between (**a**) polymer surfactants or (**b**) metal surfaces, and kerogen over time.

The interaction energy between molecules is an important influencing factor in chemical reactions. The higher the interaction energy between two groups of molecules, the greater the possibility of a chemical reaction. Figure 11 shows the changes in the interaction energy between the PS and metal surfaces, and between the PS and kerogen over time during the simulation process. At the beginning of the simulation, the interaction energy between the PS and the metal surface began to increase significantly, and then the metal surface was oxidized. The energy also slightly increased when the time exceeded 250 ps. However, the energy between the PS and kerogen changed rapidly at 250–300 ps due to the cleavage of kerogen into small molecules and the weakening of the interaction between kerogen and the PS.

**Figure 12:**
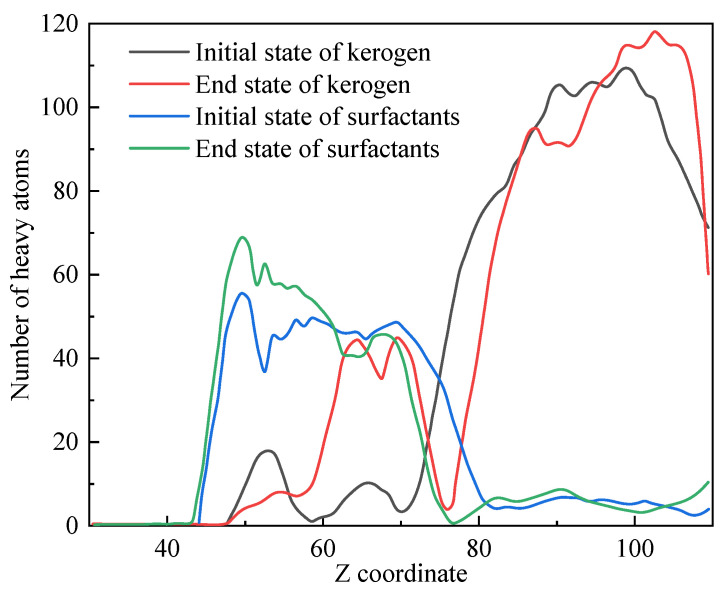
Spatial distribution of kerogen and surfactants along the *Z* axis.

Due to the periodicity of this system in the XY direction and the layered distribution of initial substances along the *Z* axis, the distribution of substances along the *Z*-axis can reflect the density changes in substances in key areas before and after the simulation process. Figure 12 shows the distribution of heavy atoms (excluding H atoms) along the *Z*-axis in kerogen and surfactants.

At the beginning of the simulation, kerogen was mainly distributed in the region of Z > 75 Å, while at the end of the simulation, a portion of the small molecules formed by kerogen cleavage transferred to the region of 60–75 Å closer to the surfactant. The distribution of surfactants shifted from 45–80 Å to 45–70 Å. This phenomenon indicated that chemical reactions mainly occurred at 75 Å and a small amount of kerogen blended with surfactants after the reaction. The vacancy of the reaction zone was due to the diffusion towards both ends of small molecules (such as C_2_–C_4_, shale oil, and C_1_ molecular fragments) that were generated immediately by the reaction.

**Figure 13:**
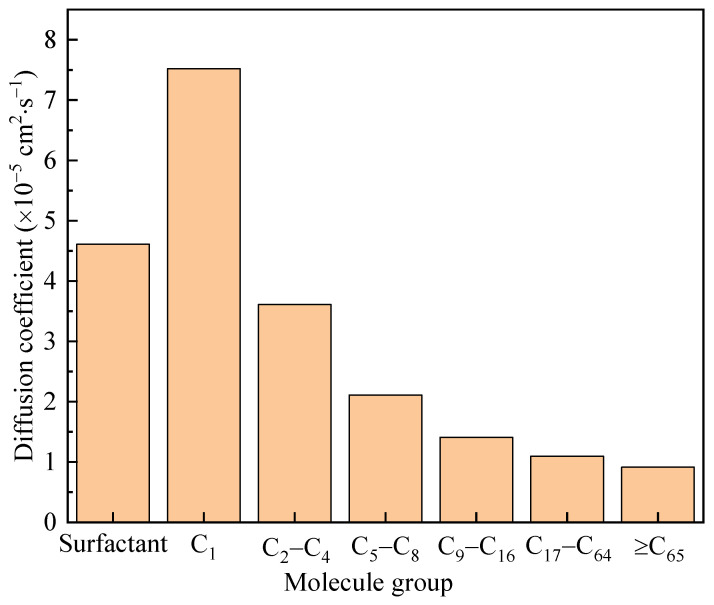
Diffusion coefficients of various molecules in the simulated system.

The diffusion coefficient measured the ability of particles to diffuse through random motion over time. In a simulation, the root mean square displacement can be obtained by grouping molecules and calculating the instantaneous displacement of each substance group in the motion trajectory, which is a key parameter for investigating particle dynamics behavior. After considering the mass of particles and the dimensions of the system, the diffusion coefficient of the system can be calculated based on the Einstein diffusion relationship. The diffusion coefficients of various molecules in this work are shown in Figure 13. The components of the surfactant used for the diffusion coefficient calculation included water and polymers. Various organic molecules were classified according to their carbon content. Small molecules such as C_1_ and C_2_–C_4_ had significantly higher diffusion coefficients than larger molecules and were prone to diffusing towards both ends. This was consistent with the conclusions presented in Figure 12.

**Figure 14:**
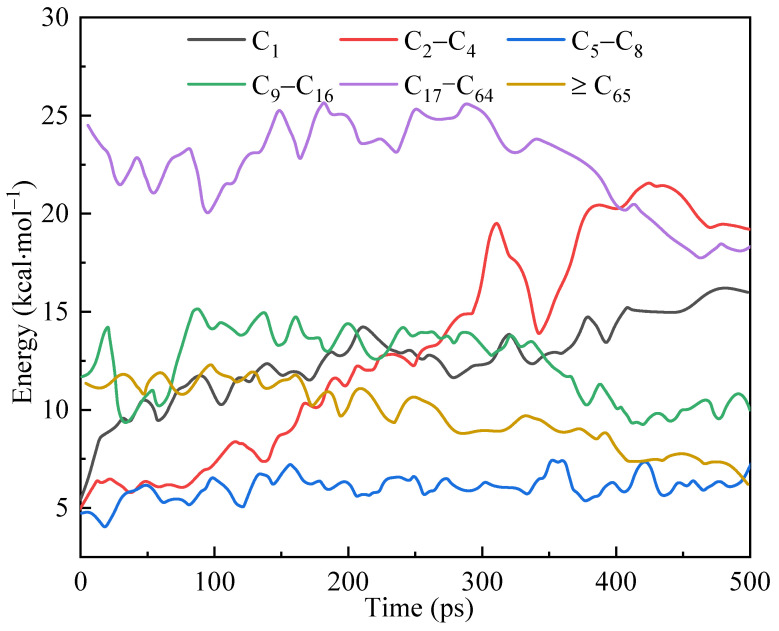
Variations in the number of various molecules in the simulation system over time.

In reaction kinetics simulations, different types of molecules can be identified and statistically analyzed to obtain information on species evolution. Figure 14 shows the various molecular quantities of kerogen molecules and their decomposition products changing with time in the simulation system. They are grouped by the number of carbon atoms.

In the early stage of the simulation, the number of macromolecules (C_65+_) decreased slowly, while the number of medium-sized molecules (C_9_–C_16_ and C_17_–C_64_) remained stable and slightly increased locally. At this time, the number of small molecules (C_2_–C_4_) significantly increased, and the number of C_1_ molecules slightly increased. This suggested that two main reactions occurred at this time: macromolecules (C_65+_) broke down from the middle, splitting into medium-sized molecules such as C_9_–C_16_ and C_17_–C_64_; the smaller side chains in the kerogen were cleaved from the main body.

In the mid-term stage of the simulation (250–300 ps), the quantities of each substance group showed inflection points, consistent with the conclusions obtained regarding thermodynamic properties and interaction energies. The number of molecules in the medium to large mass group (C_65+_, C_9_–C_16_ and C_17_–C_64_) continued to decrease from this period, while the small mass group (C_1_ and C_2_–C_4_) significantly increased. The number of molecules (C_5_–C_8_) remained constant due to the balance between the products of larger molecule fragmentation and fragmentation into smaller molecules. In addition, the generation of C_1_ and shale gas was influenced by two species changes: large molecules were gradually broken down into medium and small molecules; the cracking and detachment of large molecule side chains directly yielded smaller molecules, followed by the production of C_1_ and shale gas. The different reaction kinetics in the simulation’s early and middle stages revealed that the reaction’s mechanism had multiple different forms.

### 3.5. Cleavage Mechanism of Kerogen

**Figure 15:**
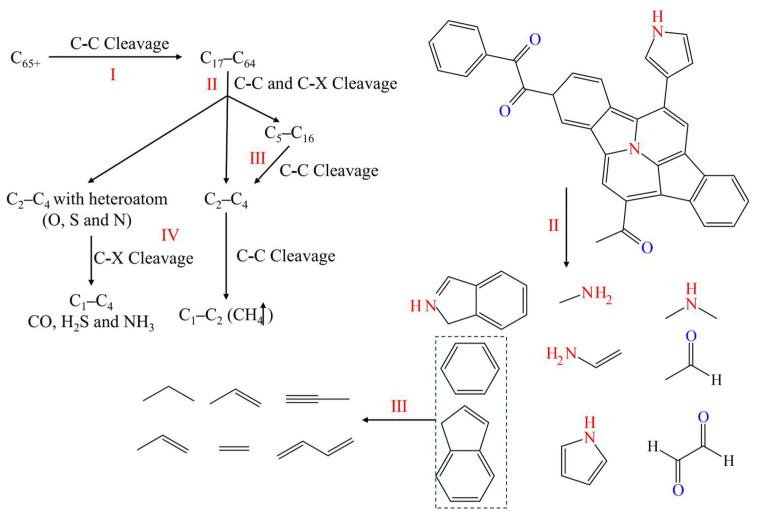
Typical pathways of kerogen cracking reaction with the I, II, III, and IV levels.

The typical reaction pathway in this simulation is obtained by analyzing simulation trajectories and classifying various reaction modes. Bond levels greater than 0.7 between atoms were considered to be formed bonds. Based on the connectivity of consecutively bonded atoms, they were considered to be molecules and molecular fragments. The typical reaction pathways were summarized according to the graph theory method for establishing reaction networks, as shown in Figure 15.

The reactions were classified into four levels (I, II, III, and IV) based on the molecular size of reactants and products. For the first-level reaction (I), the C-C bond between macromolecules (C_65+_) was cleaved, producing medium to large-sized molecules (C_17_–C_64_). The breaking of such bonds usually did not involve ring-opening reactions but rather the cleavage of weak single or double bonds between two larger constituent units.

The second-level reaction (II) involved the cleavage of C-C bonds from C_17_–C_64_ to C_5_–C_16_ molecules, as well as the cleavage of C-C and C-X (X represents O, N, S) bonds on the side chains. The directly producing molecules include C_2_–C_4_ and C_2_–C_4_ with heteroatom. This type of reaction included ring-opening reactions and non-ring-opening bond cleavage. The former, such as the cleavage of polycyclic aromatic hydrocarbons into linear molecules, mainly involves the premature cleavage of side chains, especially those containing heteroatoms (O, N, and S).

The third-level reaction (III) included the cracking of intermediate molecules (C_5_–C_16_) into small molecules (C_2_–C_4_), with most of the reactions being ring-opening reactions, which involved the process of cyclic aromatic hydrocarbons or simple aromatic hydrocarbons opening into linear fatty hydrocarbons.

The fourth-level reaction (IV) directly involved the generation of C_1_ and shale gas, and its reactants C_2_–C_4_ and C_2_–C_4_ with heteroatom from side chain cracking in the second-level reaction or ring-opening cracking in the third-level reaction. The fourth-level reaction also included the generation of inorganic substances from heteroatoms and the detachment of carbonyl groups in small molecules containing carbonyl groups to form CO. In addition, S and N atoms were ultimately converted into H_2_S and NH_3_ molecules as by-products of the reaction.

## 4. Conclusions

Surfactants are sensitive to high temperatures, resulting in a decrease in their activity or even failure, and their effect on kerogen pyrolysis has rarely been researched. A polymeric surfactant (PS) with high temperature resistance was synthesized through emulsion polymerization; it has a low molecular weight, with an MW value of 124,634. The PS had good thermal ability and its main decomposition temperature was above 300 °C. The PS enabled a reduction in the surface tension of water to less than 25 mN·m^−1^ at 25–150 °C in water, acid, or alkali solution, and slats enhanced its surface activity. The core could be transformed from an oil-wet state to a water-wet state and its oil saturation dropped by half after soaking in the PS solution, suggesting that the PS has a good cleaning effect on the shale oil reservoir. A model of the PS and kerogen was constructed using the molecular dynamics method. Under the friction of drill bits or pipes, the interface between the kerogen and the surfactant enabled the kerogen molecules to break down into smaller molecules, including methane and shale oil. The kinetics cleavage included the step-by-step cleavage process of macromolecules and side chain cleavage. The PS was shown to enhance oil recovery and promote the pyrolysis of kerogen, demonstrating promise for application in shale oil exploration and development.

## Figures and Tables

**Table 1:** Mineralogical composition of the rock sample.

Mineral Composition		Content (wt%)
Quartz		36.2
K feldspar		1.6
Plagioclase		7.2
Dolomite		2.0
Pyrite		2.2
Calcite		3.6
Amorphous		26.6
Clays	Illite	16.0
	Chlorite	3.0
	Illite/smectite (I/S)	1.6

**Table 2:** Results of core oil saturation.

Index	Data
Weight of original core (g)	53.92
Semaphore of the original core	9263.89
Weight of core with saturated oil (g)	54.99
Semaphore of the core with saturated oil	21,337.77
Oil saturation of core (%)	56.68
Semaphore of core with saturated oil soaked in the PS solution	15,867.7
Oil removal semaphore amount of PS	5470.08
Oil saturation of core after soaking in the PS solution (%)	25.63

## Data Availability

Data are contained within the article.
